# Obstacles and opportunities for the future of genomic medicine

**DOI:** 10.1002/mgg3.78

**Published:** 2014-05-06

**Authors:** Benjamin D Solomon

**Affiliations:** 1 Division of Medical Genomics, Inova Translational Medicine Institute, Inova Health SystemFalls Church, Virginia; 2 Department of Pediatrics, Inova Children's Hospital, Inova Health SystemFalls Church, Virginia

The pace of the advancement of genomic sequencing over the last decade – especially within the last 5 years – has been astounding. What initially required billions of dollars and hundreds of thousands of person-hours spread out over dozens of institutes in multiple countries can now be accomplished more accurately by one person over the course of a week, for the cost of about a thousand dollars (Lander et al. 2001; Hayden 2014). And concerns about the “thousand dollar genome” requiring an analysis costing hundreds of times that are being supplanted by the ability to leverage existing clinical/research infrastructures and through improved pipelines (Dewey et al. 2014). These pipelines include commercial offerings that allow rapid and highly sophisticated analyses based on proprietary literature curation and private variant databases (Mardis 2010).

Furthermore, the accuracy and coverage of genomic sequencing, as well as analysis capabilities, will (and must) continue to improve – for both, competition is yielding rapid progress. These gains are revealing many novel causes of human disease, including rare Mendelian disorders as well as the molecular underpinnings and genetic susceptibilities involved in complex and multifactorial conditions (Boycott et al. 2013). In the last approximately 1 year, of the over 150 individual genes newly implicated in Mendelian disorders (Solomon et al. 2013), almost three-quarters were identified through whole-exome or whole-genome sequencing. Even more impressively, in about two thirds of those genomic studies, preliminary steps (e.g., traditional homozygosity mapping or linkage studies) were not required for identification of the involved gene.

However, based on current knowledge, the returns from using cutting-edge genomics for individual patient (especially those unaffected by a rare and severe condition) become asymptotic (Dewey et al. 2014). That is, in terms of applying genomic sequencing to “genomic medicine,” with the potential to benefit virtually any individual, there remain two large general categories of obstacles. In this commentary, I will focus more on the first of these categories, although the second is no less relevant.

First, beyond questions regarding sequencing accuracy (Dewey et al. 2014), the meaning of a great deal of relatively easily identifiable genomic information is unclear, and the knowledge base is in flux. A common adage, often ominously pronounced to bright-eyed medical school students (and variably attributed and delivered) is the saying that, “half of what's in your medical textbooks is wrong – we just don't know which half.” Putting aside the fact that textbooks themselves are undergoing their own extinction event, this rather glib generalization is sobering when applied to our ability to interpret genomic (and related) data (Solomon 2014a). Our extremely incomplete knowledge of the clinical effects of genomic variation involves both the relatively more explored, but smaller portion of the genome corresponding to the coding regions of genes, as well as the large proportion of the genome that was previously relegated to “junk DNA” left over from evolution. For example, the nonexonic portions of the genome have been relatively recently suggested to have much more biological relevance and activity than previously thought (Consortium et al. 2012; Niu and Jiang 2013).

One criticism that springs from the fact that genomic knowledge is constantly growing and shifting is the idea that we as newly (self?) anointed “genomicists” are becoming lost in the seductive ability to generate enormous amounts of data, even if we cannot possibly interpret the information we are making available. In other words, now that we can rapidly sequence the whole genome, are we just getting better at quickly producing volumes of what amounts to currently opaque gibberish? In rare and severe diseases, this is clearly not the case (Boycott et al. 2013; Yang et al. 2013), but can genomics also carry over to more common medical situations that would not have traditionally warranted the involvement of a clinical geneticist? (Feero 2014).

In a sense, this criticism is a reminder of the need for traditional laboratory-based inquiries that can provide biologic Rosetta stones to better decode a person's genome and put this information into clinical context. Bioinformatic investigations based on in silico predictions using limited data can only go so far, and this limitation becomes especially important when genomic analysis attempts to provide clinically relevant information on the individual level (Solomon 2014b). Going back to basics in terms of focused and time-intensive laboratory-based studies may provide much greater clarity about the functional relevance of individual variants, as has been recently and elegantly shown (Sosnay et al. 2013) in the case of cystic fibrosis, a well-characterized genetic disease for which the cause has been known for decades (Rommens et al. 1989).

However, I would humbly assert that while traditional bench-based work attempting to characterize the functional effects of variants is more important than ever, it should be clear that it is simultaneously necessary to generate a critical mass of genomic (and related biological and clinical) data in order to move the knowledge base forward by allowing both hypothesis-driven and hypothesis-free inquiries.

This need for more large-scale genomic research applies to finding novel causes for complex and multifactorial diseases, as well as better understanding the clinical impact of individual variants already described as affecting human health. Many studies have shown that each human genome contains numerous variants that, based on current understanding, could have clinical significance (Ashley et al. 2010; Lupski et al. 2010; Solomon et al. 2012; Xue et al. 2012; Dewey et al. 2014). However, new genomic methods are also making it clear that much of the knowledge of the causes of genetic disease need to be re-evaluated (Piton et al. 2013).

Expanding the use of genomic methodologies will also shed more light on the clinical impact of genetic variants. For example, one advantage of the surge of genomic sequencing is less selection bias in terms of who is sequenced. Imagine a condition that is suspected to be caused by mutations in a certain gene. In the past, in order to test the hypothesis that mutations in that gene cause the condition, many similar patients might have had just that particular gene sequenced. Assuming the hypothesis (that mutations in that gene cause the condition) is supported, it follows that the associated phenotype will be relatively narrow – after all, only those patients were tested. With newer genomic methods, and by increasing the number and clinical range of sequenced individuals, we will be able to better question previously reported genetic causes of disease, determine new estimates of penetrance for well-described conditions, try to analyze modifying factors that modulate clinical sequelae, and better understand the range and type of manifestations related to genetic diseases (Emond et al. 2012; Johnston et al. 2012; Gonsalves et al. 2013).

**Figure 1:**
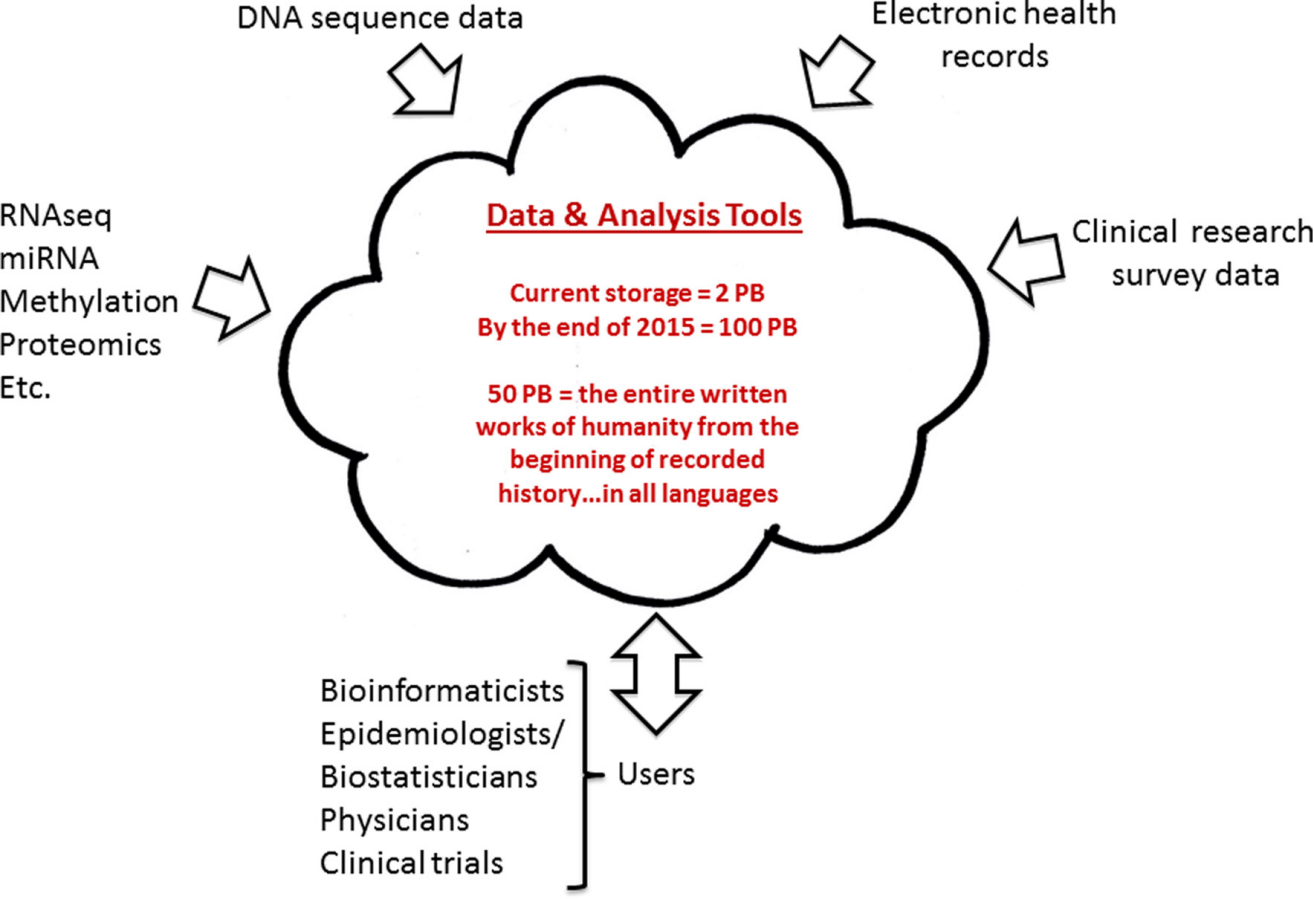
Our genomic studies at the Inova Translational Medicine Institute (the amount of storage is given in the center of the figure) focus on cloud-based storage and analysis – as well as on-premise capabilities – that integrate DNA-based data with other comprehensive phenotypic and other biologic information. We have developed a robust information technology (IT) infrastructure to enhance data storage, movement, and analysis relevant to a diverse group of end users. These types of IT considerations are increasingly important in current large-scale genomic studies, the requirements of which are shifting data handling techniques away from individually maintained datasets that can be combined on an ad hoc basis. miRNA, microRNA; PB, petabytes; RNAseq, RNA sequencing (whole transcriptome).

**Figure 2:**
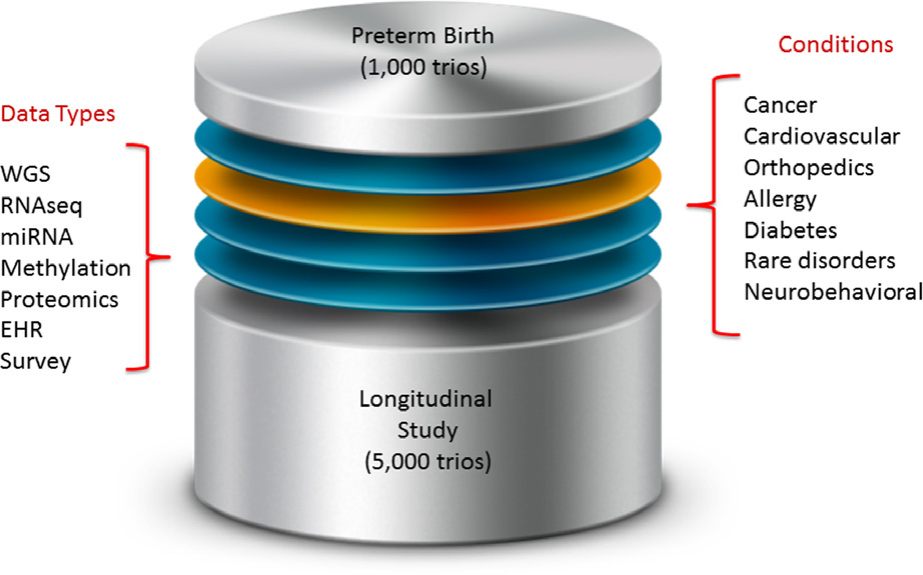
Complementary to the above figure, this figure demonstrates the combination of multiple data types arising from specific different studies. The conditions shown (as well as the approximate proportional size of each study) refer to Inova Translational Medicine Institute research initiatives, with the numbers beneath the largest studies (preterm birth and the longitudinal study) referring to the approximate number of trios. Increased statistical power and more comprehensive reference genomes can be constructed through multiple datasets, especially when detailed and standardized phenotypic annotations are available. EHR, electronic health record; miRNA, microRNA; RNAseq, RNA sequencing (whole transcriptome); WGS, whole-genome sequencing.

A number of research institutes and groups are conducting large-scale work leveraging genomic methods to better understand human health and disease. For our part, in order to address these types of questions, as well as achieve related goals involving how to best integrate genomics into medical practice, our research institute (the Inova Translational Medicine Institute) is conducting a longitudinal study focusing on trio-based whole genome sequencing. In this study, families are typically enrolled in the second trimester of pregnancy, and are followed longitudinally in what we like to term a “genomic Framingham heart study.” We currently have approximately 1200 families enrolled (80–100 new families enroll per month), with an initial goal of 5000 families, though we recognize that a larger cohort may be required to accomplish many scientific objectives. We anticipate that by marrying in-depth genomic analyses (and related biological inquiries examining RNA, protein, and epigenetic data) with comprehensive clinical information, which we collect through a single electronic health record system supplemented by study-specific surveys, we will be able to investigate the genetic and genomic contributions to many processes affecting human health, as well as test and implement optimal methods for efficient and accurate genomic interpretation and use. Additionally, we are able to augment statistical power with similarly-designed trio-based whole-genome sequencing studies at our institute; these studies focus on revealing the causes of specific disorders, such as preterm birth, diabetes mellitus, and congenital anomalies. To date, our studies have amassed over 5000 whole genome sequences with corresponding phenotypic and other biological data, and we anticipate that this number will rise to approximately 20,000–30,000 within the next several years (Figs. 1, 2).

This leads us to a second major area of obstacles: even if the effects of each genetic variant were currently understood (let alone in combination with numerous other genetic and nongenetic factors), there is the enormous and equally important question regarding how to deliver vast amounts of data seamlessly into the medical care system – that is, how to leverage genomics in a way that will benefit rather than overwhelm patients and healthcare practitioners. Again, we hope that the above-mentioned study and others like it will help determine best practices to address this issue. While the cost-benefit analysis of whole-genome sequencing is an important topic, “the horse has left the barn” – the plummeting cost, ease of access, and growing commercial opportunities virtually ensures that whole-genome sequencing will be increasingly obtainable in many clinical contexts (Feero 2014). At this point, questions have quickly shifted from “is this possible?” to “should this be done?” to “how do we best manage this?” There are multiple opinions about the best way to move forward. Some tout genomic medicine centers, while others subscribe to the idea that genomics will simply increasingly permeate the existing medical infrastructure without a need for a more dramatic paradigm shift (Francke 2013; Korf 2013; Manolio et al. 2013).

Without pretending to address this enormous topic, my experience with our own large-scale clinical whole-genome sequencing endeavors has demonstrated that there are a few key ingredients, in addition to the issues described above. Many of these ingredients are very logical, but are less frequently mentioned relative to their importance. Furthermore, if not established proactively and well, the effects may hamstring many promising approaches.

First, bioinformaticists clearly play a central role in clinically oriented analysis (Hennekam and Biesecker 2012) and clinical geneticists (or genomicists, if preferred) and genetic counselors may act as a very visible “tip of the iceberg” in terms of interfacing with patients and other practitioners. However, beneath the surface, a carefully constructed, robust information technology (IT) network is critical, even though this might not be immediately apparent to patients or clinicians, at least when functioning properly. And while this point may seem obvious, very few research enterprises, academic systems, or healthcare networks have a robust and flexible network established, and instead rely on older technologies that may have functioned reasonably well in the past. New IT systems, which will be increasingly cloud based, will involve capabilities geared towards big data genomics. These capabilities include data storage, movement, and sharing to allow nimble analysis (all done in a manner adhering to high legal and ethical standards of security and privacy); communication with the electronic health record systems and other data sources (Tarczy-Hornoch et al. 2013); (related to the previous point) compilation of genomic, other biological, and clinical data in order to allow sophisticated inquiry by multiple users.

Second, as described above, there must be clear and efficient workflow that moves from initial sample collection through result confirmation and clinical follow-up. Ideally, this can all occur without interrupting or overloading busy healthcare systems and individuals, many of whom have relatively little training and experience in genetics or genomics (Korf 2013). Most healthcare systems are already strained in terms of resources and the demands on providers. Adding these new requirements to the workload will undoubtedly be met with resistance, especially given challenges related to genomic education (Korf 2013). This would argue for a “genomics unit” of some sort – especially in the early days of genomic medicine – in order to help guide challenging situations, enhance access to genomic technologies in appropriate circumstances, and act as a lifeline in especially complex situations.

Third, along these lines, accomplishing the above two tasks in any robust fashion requires significant institutional commitment and foresight, including financial support. Even with rapidly falling sequencing prices, the type of infrastructure requirements (e.g., “simple” data storage needs) are currently too large for even a generous collection of traditional grants. And while the field of genomic medicine offers tantalizing potential in terms of cost-effective patient care, we are still in the early stages of the journey, where much work and investment are required prior to being able to capitalize on that potential.

Leaving behind the discussion on the many challenges involved, I would like to conclude by expressing my optimism about the potential for genomic medicine. In many respects, there is not a clear path forward, which is a large reason for all the excitement and hullabaloo, but finding good paths will be intellectually invigorating and rewarding. It is an incredible time to be involved in just about any aspect of clinically oriented genetics and genomics. The available tools and technologies make it possible to ask (and sometimes answer!) many questions that would have been unfathomable a few short years ago. I feel very strongly that recent advances in genomics will dramatically alter health care, and will do so in a way that will benefit individual patients and society as a whole. I also expect that this will happen more quickly than many would predict, and eagerly anticipate being involved as dreams and ideas become reality.
